# An oblique muscle hematoma as a rare cause of severe abdominal pain: a case report

**DOI:** 10.1186/1756-0500-6-18

**Published:** 2013-01-18

**Authors:** Masanori Shimodaira, Tomohiro Kitano, Minoru Kibata, Kumiko Shirahata

**Affiliations:** 1 Department of General Medicine, Iida Municipal Hospital, 438 Yawata-machi, Iida, Nagano-ken, 395-8502, Japan; 2 Junior resident, Iida Municipal Hospital, 438 Yawata-machi, Iida, Nagano-ken, 395-8502, Japan

**Keywords:** Abdominal pain, Abdominal muscle, Oblique muscle, Hematoma

## Abstract

**Background:**

Abdominal wall hematomas are an uncommon cause of acute abdominal pain and are often misdiagnosed. They are more common in elderly individuals, particularly in those under anticoagulant therapy. Most abdominal wall hematomas occur in the rectus sheath, and hematomas within the oblique muscle are very rare and are poorly described in the literature. Here we report the case of an oblique muscle hematoma in a middle-aged patient who was not under anticoagulant therapy.

**Case presentation:**

A 42-year-old Japanese man presented with a painful, enlarging, lateral abdominal wall mass, which appeared after playing baseball. Abdominal computed tomography and ultrasonography showed a large soft tissue mass located in the patient’s left internal oblique muscle. A diagnosis of a lateral oblique muscle hematoma was made and the patient was treated conservatively.

**Conclusion:**

Physicians should consider an oblique muscle hematoma during the initial differential diagnosis of pain in the lateral abdominal wall even in the absence of anticoagulant therapy or trauma.

## Background

Abdominal wall hematomas are an uncommon cause of acute abdominal pain and are often misdiagnosed. They result from rupture of the epigastric vessels or the deep circumflex iliac artery (rarely), or from tears of the fibers of the rectus abdominis or lateral oblique muscles [1,2]. They may occur because of trauma, physical exercise, recent surgery, or injection procedures. They may also occur because of increased intraabdominal pressure from coughing, sneezing, vomiting, or straining during urination, defecation, and labor [1,3-6]. Other predisposing factors include increased age, arterial hypertension, atherosclerosis, and systemic anticoagulant therapy. Most abdominal wall hematomas occur in the rectus sheath, and a hematoma within the oblique muscle is very rare. Here we report a case of an oblique muscle hematoma in a middle-aged patient.

## Case presentation

A 42-year-old man presented with a painful, enlarging, lateral abdominal wall mass, which appeared after playing baseball. He gave no history of direct abdominal trauma such as collision with another player. He had a history of hyperuricemia; however, he had not undergone any therapy for the same. There was no family history of bleeding diathesis or hematological diseases. On physical examination, his vital signs were as follows: temperature 36.8°C, blood pressure 108/69 mm Hg, pulse rate 58 beats/min, and respiratory rate 12 breaths/min. A firm mass was palpable over the left lateral abdominal wall and it was tender. The skin color of the area was normal. Blood biochemistry on laboratory examination was within normal ranges and the platelet count was 174 ×10^3^ cells/μl, prothrombin time was 11.8 s, international normalized ratio was 0.99, and activated partial thromboplastin time was 32.6 s.

**Figure 1:**
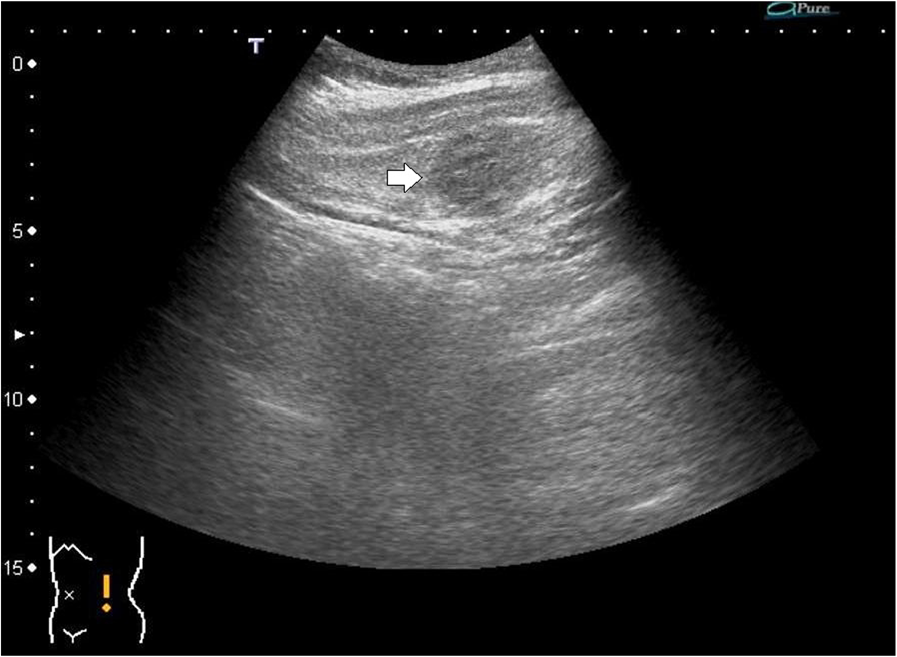
**Abdominal ultrasonography (US).** An abdominal US showed a heteroechoic mass (white arrow) in the patient’s left lateral abdominal wall.

**Figure 2:**
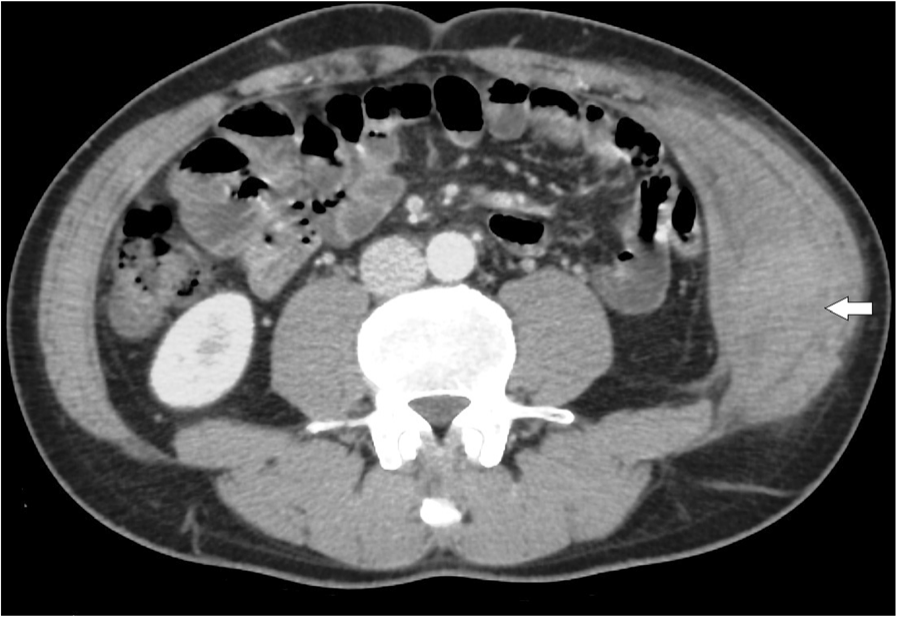
**Contrast-enhanced CT.** Contrast-enhanced CT did not show extravasation of the contrast material within the mass (white arrow).

**Figure 3:**
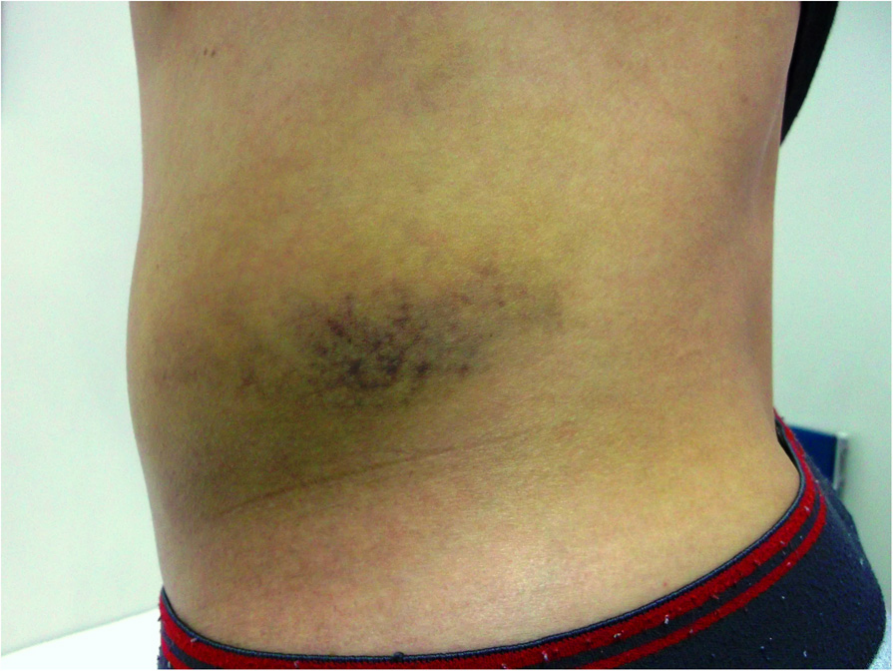
**Ecchymosis on the left lateral abdomen.** Ecchymosis presented 4 days later on the patient’s left lateral abdomen.

Ultrasonography (US) showed a heteroechoic, well-defined mass in the patient’s left lateral abdominal wall (Figure 1). An emergency noncontrast computed tomography (CT) of the abdomen showed a hyperdense mass in the left internal oblique muscle measuring 10.5 × 6.5 × 5.5 cm. Contrast-enhanced CT did not show extravasation of the contrast material within the mass (Figure 2). The diagnosis of an oblique muscle hematoma was made. The patient was conservatively treated with analgesics. Four days after the first visit, the patient revisited our department for follow-up. His abdominal pain improved, but an ecchymosis was observed on the patient’s left lateral abdominal skin (Figure 3).

## Discussion

Abdominal wall hematomas are one of the causes of acute abdominal pain. A rectus sheath hematoma caused by the rupture of the epigastric artery is a rare, but well-described, manifestation of abdominal hematoma. On the other hand, an oblique muscle hematoma caused by a rupture of the deep circumflex iliac artery is very rare [1]. The most common presenting signs and symptoms of these hematomas are acute abdominal pain and firm, palpable abdominal wall masses. Because of their rarity, abdominal wall hematomas can be mistaken for several common acute abdominal conditions such as appendicitis, sigmoid diverticulitis, perforated ulcers, ovarian cyst torsion, tumors, or incarcerated inguinal hernias [7]. Misdiagnosis may lead to unnecessary negative laparotomies with increased morbidity and mortality [3]. Therefore, these diseases should be considered as differential diagnoses.

Many risk factors have been reported for abdominal wall hematomas. These include aging, anticoagulant therapy, platelet disorders, trauma, recent surgery, injection procedures, and physical exercise as well as increased intraabdominal pressure from coughing, sneezing, vomiting, or straining during urination, defecation, or labor [6]. In the review of 126 cases of rectus sheath hematoma, it is reported that most patients (69%) were on some forms of anticoagulation therapy and the mean age was 67.9 years [5]. Our patient was not taking any medications affecting blood coagulation and laboratory data regarding coagulation function were within normal limits. Therefore, his internal oblique muscle was considered to be injured by a sudden or repetitive trunk movement, either rotation or flexion/extension, while playing baseball [8].

An abdominal wall mass with ecchymosis is the most important diagnostic finding for suspicion of a hematoma. However, abdominal wall ecchymosis is a late sign and the average time between its presentation and its onset takes about 4 days, as reported in the literature [9]. Furthermore, ecchymosis is a rare presentation for an abdominal wall hematoma. A study by Cherry et al. showed that only 17% abdominal wall hematoma patients present with an abdominal wall ecchymosis [5]. In our patient, ecchymosis was detected 4 days after the appearance of the abdominal wall hematoma. To the best of our knowledge, this is the first report of an oblique muscle hematoma that was accompanied by ecchymosis.

The diagnosis of an oblique muscle hematoma is made by combining medical history, laboratory examination findings, and US and/or radiological findings. US and CT scans can provide useful information for differential diagnosis to avoid unnecessary surgery [6]. US can be useful as a first-line investigation because it is widely available and portable [10]. In addition to US, contrast-enhanced CT can detect and evaluate active bleeding from the rupture site [4]. In the present case, contrast-enhanced CT findings did not show active bleeding. Therefore, we could not confirm which artery was ruptured by CT findings. Even in a patient without contrast extravasation at the bleeding site as observed on CT, selective digital subtraction angiography could be a useful imaging technique to identify an active bleeding point [11].

Although there is no grading for an oblique muscle hematoma because of its rarity, the following grading system has been established for a rectus sheath hematoma on the basis of CT findings. Grade I is an intramuscular hematoma with an observable increase in muscle size. Grade II is also an intramuscular hematoma but with blood between the muscle and transversalis fascia. Grade III hematoma may or may not affect the muscle and blood is seen between the transversalis fascia and muscle in the peritoneum and prevesical space that results in a drop in hemoglobin [12]. Grade I hematoma may resolve rapidly within approximately 30 days, whereas Grade II hematomas require 2–4 months and Grade III hematomas require more than 3 months to resolve [12]. Hence, a classification based on CT findings could help a physician in predicting a patient’s outcome.

Conservative treatment including bed rest and analgesics are appropriate in most patients with abdominal wall hematomas. Although most are self-limiting because the bleeding usually stops without intervention, some patients show significant morbidity and the overall mortality rate is reported to be 4%. Surgical intervention or transcatheter arterial embolization is recommended when conservative management fails [1,4]. In our case, conservative treatment was administered because CT findings did not suggest active bleeding.

## Conclusion

An oblique muscle hematoma is a very rare condition. In the clinical setting, abdominal US and contrast-enhanced CT are useful for a diagnosis. A correct diagnosis is important to avoid increasing morbidity or unnecessary surgical intervention. Treatment is mainly conservative and includes pain management. Physicians should consider an oblique muscle hematoma in the initial differential diagnosis of abdominal pain even in the absence of history of anticoagulant therapy or obvious trauma.

## Consent

Written informed consent was obtained from the patient for publication of this case report and any accompanying images. A copy of the written consent is available for review by the Editor-in-Chief of this journal.

## Competing interests

The authors declare that they have no competing interests.

## Authors’ contributions

MS wrote the manuscript. TK contributed to the diagnosis and revised the manuscript. MK and KS reviewed the literature. All authors contributed intellectual content, have read and approved the final manuscript.
